# Perceived mathematical ability under challenge: a longitudinal perspective on sex segregation among STEM degree fields

**DOI:** 10.3389/fpsyg.2015.00530

**Published:** 2015-06-09

**Authors:** Samantha Nix, Lara Perez-Felkner, Kirby Thomas

**Affiliations:** ^1^Department of Educational Leadership & Policy Studies, Florida State UniversityTallahassee, FL, USA; ^2^Department of Sociology, Florida State UniversityTallahassee, FL, USA

**Keywords:** higher education, gender, STEM, pipeline, perceived ability, ability-related beliefs, college major

## Abstract

Students' perceptions of their mathematics ability vary by gender and seem to influence science, technology, engineering, and mathematics (STEM) degree choice. Related, students' perceptions during academic difficulty are increasingly studied in educational psychology, suggesting a link between such perceptions and task persistence. Despite interest in examining the gender disparities in STEM, these concepts have not been considered in tandem. In this manuscript, we investigate how *perceived ability under challenge*—in particular in mathematics domains—influences entry into the most sex-segregated and mathematics-intensive undergraduate degrees: physics, engineering, mathematics, and computer science (PEMC). Using nationally representative Education Longitudinal Study of 2002 (ELS) data, we estimate the influence of perceived ability under challenging conditions on advanced high school science course taking, selection of an intended STEM major, and specific major type 2 years after high school. Demonstrating the importance of specificity when discussing how gender influences STEM career pathways, the intersecting effects of gender and perceived ability under mathematics challenge were distinct for each scientific major category. Perceived ability under challenge in secondary school varied by gender, and was highly predictive of selecting PEMC and health sciences majors. Notably, women's 12th grade perceptions of their ability under mathematics challenge increased their probability of selecting PEMC majors over and above biology. In addition, gender moderated the effect of growth mindset on students' selection of health science majors. Perceptions of ability under challenge in general and verbal domains also influenced retention in and declaration of certain STEM majors. The implications of these results are discussed, with particular attention to access to advanced scientific coursework in high school and interventions aimed at enhancing young women's perceptions of their ability, in particular in response to the potentially inhibiting influence of stereotype threat on their pathways to scientific degrees.

## Introduction

Socially influenced beliefs about mathematics ability have been studied as possible explanations for the gender gap in science, technology, engineering, and mathematics (STEM) higher education. Nevertheless, there remains insufficient conceptual and empirical clarity about how beliefs influence gendered differences over time, specifically during upper secondary and post-secondary school—the primary years for attrition from pathways to science careers (Berryman, 1983; Morgan et al., 2013). Notably, theories have emerged suggesting that persistence when encountering potentially negative or challenging situations is influenced by students' perceived ability to complete specific tasks (*self-efficacy*) (Bandura, 1977; Pajares, 1996), beliefs about the malleability of their abilities (*mindset*) (Dweck, 2007, 2008), the alignment of their skills to the challenge presented by the material (*flow*) (Csíkszentmihályi and Csikszentmihályi, 1988; Shernoff et al., 2003), and fear of confirming negative stereotypes related to their identities (*stereotype threat*) (Steele, 1997; Beilock, 2008). Related, students' self-assessments of their mathematics ability appear to vary by gender and influence STEM degree choice (Correll, 2001; Parker et al., 2012; Perez-Felkner et al., 2012). These studies indicate a growing interest in examining the puzzling persistence of gender disparities in STEM. These concepts have not been considered in tandem however, to investigate how domain-specific and domain-general *perceived ability under challenging conditions* influence the gender gap in the most sex-segregated undergraduate degrees: physics, engineering, mathematics, and computer science (PEMC).

This study takes on this gap in the literature. Using the nationally representative Education Longitudinal Study of 2002 (ELS) data, we estimate the influence of mindset and self-perceptions of mathematics ability in challenging contexts on each subsequent step in the STEM pipeline: completing advanced high school science courses, persistence in a STEM major, and specific STEM major selection. Importantly, we compare and control for variation in students' response to challenge in verbal and mathematics tasks, while also controlling for more objective measures of verbal and mathematics ability. Moreover, this study uses the most recent and complete U.S. panel data available to examine how perceptions of mathematics ability on difficult tasks changes over time^1^, during the years that appear to be when most girls who exit the STEM pipeline conclude that they are more capable in other domains.

## Previous research

Empirical studies demonstrate a persistent gender gap in post-secondary degree attainment in certain mathematically-intensive STEM disciplines, both internationally (OECD, 2013) and domestically (NSF, 2013). Students' perceptions in response to challenges and negative feedback may be particularly informative to enhancing our understanding of how to encourage women's persistence in these fields. Performance feedback is formally given to students through grades, which some have suggested can imply subject-field difficulty to students (Drew, 2011; Putman et al., 2014). Research on the influence of STEM grades is mixed, however. For instance, in his longitudinal study of a single, elite research institution, Ost (2010) found that female physical science majors were more likely than their male physical sciences or female life sciences counterparts to change majors in response to lower grades in their STEM courses. In contrast, Griffith's (2010) findings from an analysis of multi-institutional datasets suggests that the positive effects of higher STEM GPAs on STEM persistence is likely more important for men. Such findings have led some scholars to conclude that grades cannot adequately predict students' responses to challenge, and instead suggest investigations of social psychological factors that may play an even larger role in student choice-making processes (Rask, 2010; Stearns et al., 2013).

This study builds upon these efforts by looking specifically at the role of beliefs about difficult mathematics material, a vital competency area for success in postsecondary STEM fields. To frame our study, we discuss factors that have been shown to impact persistence in scientific fields. In particular, we focus on self-perceptions of ability in mathematics with difficult material, from tenth grade through university major selection.

### How demographic, academic, and schooling contexts influence scientific ambitions

Previous scholars have demonstrated links between family background, high school preparation, and environmental factors that have played a role in students' decisions to pursue degrees in scientific fields. Overall, female gender has been widely shown to differentially affect youths' preparedness for and persistence in certain STEM fields, both across and within racial-ethnic groups. In a qualitative study of prospective STEM majors at seven campuses in the early 1990s, Seymour (1999) found that women who entered college as potential STEM majors were less rigid in their choice of major than were men, with the exception of those most socioeconomically disadvantaged. Interestingly, Hanson (2008) finds that contemporary labor norms in the black community contribute to black girls' resilience in pursuing scientific careers. Moreover, black and Latina girls seem to take more advanced high school mathematics course sequences than their male peers (Riegle-Crumb, 2006). In a study of Latino STEM majors, Cole and Espinoza (2008) found that participants' gender had the third largest positive impact on GPA, providing evidence that Latinas outperform Latinos in STEM postsecondary classrooms. A national longitudinal study using ELS data similarly found a nuanced relationship between gender and race/ethnicity in who chooses STEM majors in college, with Latino males being the group least likely to pursue STEM and black males being the most likely among those who had completed pre-collegiate STEM coursework (Perez-Felkner et al., 2014).

In the later years of high school, students may elect to take advanced mathematics and science courses. Gendered patterns in completion of these courses have been found (Riegle-Crumb et al., 2006), as girls may be less inclined to pursue areas that have not been associated with female success. Notably, some gaps have closed in recent years. For example, the National Center for Education Statistics reported gender parity in high school calculus completion in 2009 (Kena et al., 2014). While research on mathematics course taking is more extensive than that on science course taking (e.g., Davenport et al., 1998), the latter may be more important given the persistence of gendered patterns in science. For example, this report also found that girls were less likely to complete high school physics (33% of girls as compared to 39% of boys).

These high school course decisions can influence post-secondary STEM major selection and degree completion, in particular in PEMC fields. Across three nationally representative cohorts attending high school in the 1980s, 1990s, and 2000s, completion of physics and calculus before H.S. graduation each increased students' chances of enrolling in physical science or engineering majors in college (Riegle-Crumb et al., 2012). While completing advanced coursework increases girls' chances of going on to declare postsecondary majors in physical sciences, engineering, mathematics, and computer science, those girls who enrolled in more advanced mathematics and science coursework seemed to have more negative self-assessments of their ability and mindsets regarding mathematics ability (Perez-Felkner et al., 2012). Holding these negative beliefs may contribute to struggles women might encounter as some of the comparatively few women majoring in these fields in college. Nevertheless, this body of research suggests that advanced coursework positions students—including young women—to choose PEMC majors.

Decades of research have indicated that high school contexts contribute to variation in students' postsecondary outcomes (Coleman et al., 1982; Perez-Felkner, in press), which may influence their preparedness for and persistence in scientific majors. Geographic proximity to college may influence where and in what type of college students enroll (e.g., Rouse, 1995); Latinos are especially likely to attend college closer to home (López Turley, 2009). Proximity to college seems to influence enrollment among both advantaged and low-income students, but is less of an issue among students in the northeast, which has both a greater density of post-secondary offerings, selective colleges, and urban areas (Griffith and Rothstein, 2009). Some studies have suggested that students are less likely to select STEM majors if they attend selective postsecondary institutions (Griffith, 2010; Engberg and Wolniak, 2013), while others suggest that institutional selectivity has no effect on women and underrepresented students' pursuit of degrees in scientific fields major (Smyth and McArdle, 2004; Perez-Felkner and Schneider, 2012). At the secondary level, students attending urban schools have tended to have lower postsecondary outcomes (Niu and Tienda, 2013). Moreover, girls in rural schools were found to be more likely than those in suburban or urban schools to choose STEM majors in college, irrespective of their high school preparation for these fields (Perez-Felkner et al., 2014). Gendered differences in scientific degrees may then be partially explained by regional variation in both the density of 4-year colleges and the proportion of students living in cities vs. suburban and rural communities.

### Beliefs about mathematics

While some continue to argue that cognitive ability in mathematics varies by gender and drives the gap in the STEM labor force (Hedges and Nowell, 1995; Summers, 2005), empirical evidence largely refutes this claim (Hyde and Linn, 2006). Notably, a meta-analysis of U.S. state assessments of mathematics performance found that 2nd through 11th grade students did not significantly differ by gender; however limitations in these data did not allow for analyses of complex problem solving and advanced mathematics, areas in which extant research finds that gender differences may be more likely to emerge (Hyde et al., 2008). Research spanning two decades' of nationally representative cohorts reveals gender gaps in some STEM majors are not fully explained by achievement in mathematics (Riegle-Crumb et al., 2012). Many have theorized that individuals' understanding of themselves and mathematics can influence students' major choices. For instance, Perez-Felkner et al. (2012) examined ELS data to show that subjective orientations to mathematics (operationalized as perceived mathematics ability as well as engagement in, valuing, and mindset toward mathematics ability) was positively and significantly correlated with selection of PEMC and Biological sciences majors. Similarly, but with a focus on self-concept, Parker et al. (2012) analyzed large-scale datasets from both Germany and England. Their findings revealed that mathematics self-concept predicted students' entry into physical sciences, engineering, and mathematics. In addition, self-concept was found to be a more powerful predictor of major choice than standardized tests of ability, consistent with studies showing that ability does not explain the gender gap (Perez-Felkner and Schneider, 2012; Riegle-Crumb et al., 2012).

Correll (2001) used National Education Longitudinal Study (NELS) 1988 data to show that girls underrate their abilities in mathematics, even after controlling for performance feedback and objective measures of their abilities. Also using NELS, but extending her research into postsecondary outcomes, Ma (2011) found that perceptions of mathematics ability predicted entry into a STEM field, and that those perceptions were least predictive of entry into life science majors. Consistent with this research, Sax (1994) analyzed Cooperative Institutional Research Program (CIRP) 1985/1989 data and found that mathematics self-concept at the beginning and end of college was significantly lower for women in her sample than men. Importantly, her research also showed that for women in particular, mathematics self-rating at the end of college was significantly and strongly predicted by confidence in mathematics ability before entering postsecondary environments.

Research also reveals that perceptions do not exist in a vacuum. For instance, Correll (2001) found that students compare their progress in mathematics and verbal domains, with higher scores and perceptions of ability in English predicting lower perceptions of mathematics ability and selection out of advanced mathematics courses. Wang et al. (2013) found evidence that ability in both mathematics and verbal domains might lead women to believe that they have a wider range of career choices. In particular, those with high ability in both mathematics and verbal domains were predicted to select out of STEM fields compared to women with high mathematics ability and moderate verbal ability.

Given the findings summarized above, this study considers beliefs about abilities in general, verbal, and mathematics domains. Further, we focus particularly on students' perceived ability to overcome challenging or difficult material. We hypothesize that variations in those perceptions predict selection of advanced science courses in high school, persistence in STEM fields, and selection of mathematics-intensive majors.

## Conceptual framework

Our research questions and design respond primarily to prominent social psychological theories, which also inform the interpretation of our results.

### Self-efficacy

Bandura's (1977) self-efficacy is perhaps the most widely applied educational motivation theory, especially in investigations of the gender and race/ethnicity variation in STEM fields (Pajares, 1996; Rittmayer and Beier, 2009). Describing students' perceptions of their ability to complete specific tasks in particular domains (such as long division in mathematics), self-efficacy links beliefs, behaviors, and environments to explain students' choice making processes (Pajares, 1996; Zimmerman, 2000). The theory's value arises in part from its wide application—it can be applied across disciplines, given the application is task-specific. In focusing on one's beliefs in their ability to *do* a specific task, self-efficacy measures may miss students' immediate and overall assessment of a domain: whether or not it presents an overwhelming challenge to the student to begin with, before they start contemplating their ability to complete specific tasks within that field of study. Therefore, our analysis focuses on domain-specific (rather than task-specific) perceptions of ability under challenge.

### Flow

In contrast to self-efficacy, Csikszentmihalyi's flow theory integrates people's perceptions of challenge and their corresponding perceptions of ability. Flow theory, at its heart, is about “optimal” experience (Nakamura and Csikszentmihalyi, 2002, p. 89)—the moment when people become so involved in their tasks, that they lose their sense of self-consciousness and the passage of time. According to the theory, people arrive in this state of being when a task just meets the threshold of their abilities, and thus are perceived as challenging, but not overwhelming (Csíkszentmihályi and Schneider, 2000; Nakamura and Csikszentmihalyi, 2002). Additionally, people gain such satisfaction from moments when they are in flow, that they seek out tasks that will continue to provide them with such experiences (Csíkszentmihályi and Csikszentmihályi, 1988; Csíkszentmihályi and Csíkszentmihályi, 1991). We propose that students who believe that they can overcome challenge in mathematics domains will continue to seek those experiences out, via selecting mathematics-related majors while in college.

### Mindset theory

Dweck's (2000, 2006) mindset theory proposes that students do not have a universal response to challenge. Instead, their response to challenge is mediated by their mindset, or their belief that abilities can be developed or are innate. Those who believe that intelligence is innate—people with a fixed mindset—tend to be much less likely to select challenging tasks, because they do not want to disconfirm their intelligence in front of others. In contrast, those who believe that intelligence is malleable or can be developed—growth mindset individuals—tend to take on challenging material because they do not believe the task at hand implies anything specific about their overall intelligence. Thus, fixed mindset individuals are thought to have helpless responses to challenging material, while growth mindset individuals are thought to have mastery responses to challenging material (Dweck, 2000, 2006).

Importantly, girls have been shown to be more likely to hold a fixed mindset (Dweck, 2007), suggesting that they may implement helpless behaviors when confronting a difficult task. Further, much of the research on this topic argues that adjustments to women's and underrepresented minorities' mindsets could help with gaps in STEM participation (Dweck, 2008; Good et al., 2012; Mangels et al., 2012). If girls are more inclined to view their abilities as fixed rather than malleable, they may also be more likely to believe that they are not capable when they encounter setbacks on challenging mathematics tasks. How gender moderates perceived ability becomes particularly important, given the prevailing stereotypes that girls encounter regarding their mathematics ability.

### Stereotype threat

According to Steele (1997) stereotype threat occurs when an individual internalizes the stereotypes of a group with which they identify, such as women's perceived weakness in mathematics. Bielock and colleagues have proposed a link between stereotype threat and task success via working memory (Beilock, 2008; Rydell et al., 2009; DeCaro et al., 2010). Importantly, these studies use experimental research design to establish that women's working memory is inhibited when they are reminded about the gender stereotype that women are less successful at mathematics, and propose interventions to help mitigate that effect (Good et al., 2003). Therefore, we recognize that females' perceptions of ability to overcome challenge might be particularly important as they move into increasingly more gender-segregated academic environments while advancing toward STEM degrees, in which stereotypic beliefs may be more salient.

## Research questions and hypotheses

We build upon the previous research presented above to examine the complex interplay between gender, perceptions, and participation in STEM, particularly under difficult or challenging conditions in mathematics and other domains. Specifically, four research questions guided our research:

1. To what degree do domain-specific and domain-general perceptions of ability under challenge differ by gender?
2. What is the relationship between perceived ability under challenge in mathematics and advanced high school science course enrollment?
3. To what extent does perceived ability under challenge in mathematics predict staying in a STEM field as intended before entering postsecondary education? How is this relationship moderated by gender?
4. What is the relationship between perceived ability under challenge in mathematics and selection of mathematics-intensive science majors (PEMC), and how is that relationship moderated by gender?

**Figure 1:**
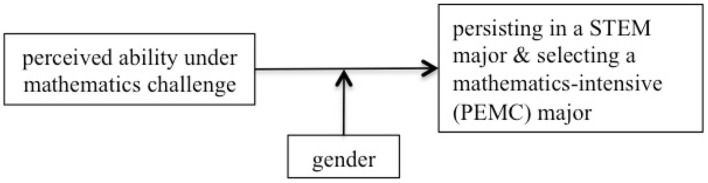
**Conceptual model of how gender moderates perceived ability and major choice**.

As emphasized in the research questions above, we hypothesize that gender moderates the relationships between perceived ability under mathematics challenge and outcomes for subsequent steps in the STEM pipeline. Therefore, our research questions build upon one another, leading to our primary focus: an examination of the relationships between perceived ability under challenge, gender, and selection of mathematics-intensive majors (see Figure 1).

## Methods

### Data source and participants

We used nationally representative Education Longitudinal Study (ELS) panel data to address our research questions. Collected by the National Center for Education Statistics, probability sampling was implemented for the base year data collection effort in 2002, yielding a sample of 17,591 eligible 10th graders from 752 high schools across the United States. Parents, administrators, staff, and teachers were also surveyed. Follow-ups were then conducted in 2004 (during most students' 12th grade year), 2006, and 2012 (Ingels et al., 2007). For clarity, we discuss the data primarily in reference to participants' stage in education. For instance, “10th grade” refers to 2002 or base year data, “12th grade” refers to 2004 or first follow-up data, and “2 years after high school” refers to 2006 or second follow-up data. This study uses the high school (10th and 12th grades) and 2 years after high school student surveys, including some control variables (such as family income, education, and high school environment measures) gleaned from the accompanying parent and administrator surveys (see the Appendix in Supplementary Material for more details). Survey administrators reported an 88% weighted response rate for students participating in these first three waves: 10th grade (2002), 12th grade (2004), and 2 years after high school (2006) (Ingels et al., 2007).

Our analytic sample represents the college-going population of U.S. students who were tenth graders in the spring of 2002 and enrolled in college between 2004 and 2006. We include only students who attended either 2- and 4-year institutions by 2 years after high school, as our second and third research questions are related to college major choice. Any students who remained undecided or undeclared were coded as such but retained in our analyses. Therefore, of the 16,197 observations in the ELS dataset, we found that 10,534 had enrolled in a postsecondary institution by 2 years after high school. Because of our interest in race as well as gender, we additionally excluded respondents from groups with overly low representation in the sample^2^. We then used listwise deletion for any remaining missing observations on the independent and dependent variables, yielding a final analytic sample of 4450 cases. Lastly, we used response adjusted, calibrated bootstrap replicate weights (ELS variables f2byp1-f2byp200), and panel survey weighting (with f2bywt) to adjust for stratification in the sample design. Sample descriptive statistics are discussed throughout the measures section.

### Measures

#### Outcome variables

#### Science pipeline

First, we examined the most advanced science course students took in high school. We collapsed the original categories from eight to three to enhance the interpretability of our analyses, as those on the lowest end of the science pipeline tended not to attend nor complete college. Thus, the science pipeline variable focuses on the upper end of the scale and represents students' completion of three levels of science coursework: (1) chemistry I or physics I or less, (2) both chemistry I and physics I, and (3) chemistry II and physics II. Biology and other sciences were included in the science pipeline variable, but the ranking privileges chemistry and physics as indicators of having completed the “science pipeline” in high school. We report on the relationship between gender and completion of these science pipeline courses in Table 1. Fewer women participated in the highest and middle level of science coursework compared to men (Table 1). Correspondingly, there is a higher percentage of women (53.1%) who only completed the lowest level of science coursework (Chemistry I or Physics I and below) compared to men (45.4%).

**Table 1:** **Sample descriptive statistics on dependent variables**.

	**Men**		**Women**			
	**%**	**SE**	**%**	**SE**	**Min**	**Max**
**SCIENCE PIPELINE**						
Chemistry I or Physics I and below	45.4%	1.6	53.1%	1.6	0.0	100.0
Chemistry I and Physics I	26.0%	1.6	22.6%	1.3	0.0	100.0
Chemistry II and Physics II	28.6%	1.6	24.3%	1.3	0.0	100.0
**MAJOR RETENTION**						
Abstainers	68.1%	1.4	86.9%	0.8	0.0	100.0
Stayers	16.6%	1.2	3.7%	0.4	0.0	100.0
Leavers	10.1%	0.9	4.0%	0.5	0.0	100.0
Newcomers	5.3%	0.7	5.4%	0.5	0.0	100.0
**MAJOR TYPE**						
Undeclared/undecided	24.0%	1.4	20.6%	1.0	0.0	100.0
Non-STEM	39.7%	1.5	42.5%	1.3	0.0	100.0
PEMC	17.1%	1.2	4.0%	0.5	0.0	100.0
Biology	4.7%	0.6	5.0%	0.5	0.0	100.0
Health	4.0%	0.6	15.9%	1.0	0.0	100.0
Social/Behavioral and other sciences	10.4%	0.9	11.9%	0.7	0.0	100.0

n = 4450 respondents from the National Center for Education Statistics' Education Longitudinal Study 2002/2006 restricted data. Student-level replicate weights particular to the base year through 2nd follow-up (2002/2006) waves were used to enhance the correspondence between sample and population results. Restricted-use NCES data requires rounding these descriptive results to the nearest tenth.

#### Major retention

Next, we were interested in what encouraged retention in STEM fields. To understand this, we compared participants' intended major to their declared major 2 years after high school. The intended major variable was retrospective, as students were asked 2 years after high school which field they intended on entering before starting their postsecondary educations. Further, due to the original coding of the intended major data, intended PEMC and biology majors could not be disaggregated (see the Appendix in Supplementary Material for more detail). Therefore, the major retention variable includes four categories: (1) abstainers (never intended or majored in PEMC and/or biology), (2) stayers (intended and majored in PEMC and/or biology), (3) leavers (intended but did not major in PEMC and/or biology), and (4) newcomers (did not intend but majored in PEMC and/or biology).

Overall, a larger proportion of men (32.0%) in our sample participated in a PEMC and/or biology major in some way, compared to women (13.1%, Table 1). A full 86.9% of women in our sample abstained from PEMC and/or biology, neither intending nor enrolling in those fields by 2 years after high school. This high lack of engagement drives the lower percentages in the other major retention categories for women in our sample. For instance, only 3.7% of women persisted in a PEMC and/or biology field as intended compared to 16.6% of men. In addition, 4.0% of women left a PEMC and/or biology field, compared to 10.1% of men. Finally, a comparable proportion of men and women (5.3 and 5.4%, respectively) were considered newcomers, entering a PEMC and/or biology field by 2 years after high school, even though it was not their intended major.

#### Major type

The last outcome variable provides information on the specific type of major students selected 2 years after leaving high school. Because of the importance of disaggregating STEM majors by fields of study (Perez-Felkner et al., 2012; Ceci et al., 2014), we looked specifically at students majoring in the physical sciences, engineering, mathematics, and computer sciences (PEMC) against other STEM majors; specifically, we compare PEMC to biology, health, social/behavioral and other sciences, and non-STEM majors. We additionally compare with undecided/undeclared students, to achieve a more representative set of analyses from high school through college. Full details (including the list of majors included in each category) are provided in the Appendix in Supplementary Material.

Looking specifically at this outcome for our sample on Table 1, we see that 2 years after high school, 24.0% of men and 20.6% of women had an undeclared or undecided major. A larger proportion of women (42.5%) had a non-STEM major, compared to men (39.7%). Consistent with the previous literature, a far smaller percentage of women majored in PEMC fields (4.0%) compared to men (17.1%), though these results are roughly mirrored when looking at health fields (15.9% of women vs. 4.0% of men). Men and women participated at comparable levels in biology fields (4.7% of men and 5.0% of women), with a slightly higher proportion of women (11.9%) declaring a social/behavioral and other science major compared to men (10.4%).

#### Perceived ability under challenge in domain-general and verbal and mathematics domains

As noted above, this study is primarily concerned with students' perceived ability under challenge. We operationalized this concept by selecting ELS items that represented students' perceptions of their ability to use mastery-oriented behavior and comfort with complex or difficult material. A brief discussion of each measure of perception of ability to overcome challenge is discussed below, with full details in the Appendix in Supplementary Material. All perceived ability under challenge variables were mean-centered for interpretability. We report mean scores for men and women in the Results Section.

#### General index

Five items from the 10th grade survey were used to assess students' perceived ability under challenge in general, as opposed to within a particular subject domain. Original scores on each ranged from 1 to 4, with higher values representing higher agreement with each statement. From these five statements, we developed a mean item index to represent domain-general perceived ability under challenge (α = 0.865).

#### Verbal index

Tenth graders were also asked to report their agreement with three statements related to their comfort with difficult verbal tasks and use of mastery behavior in that field. Similar to the items on the general index, each of these responses were originally coded 1–4; a score of 4 indicates the highest agreement with each statement. These variables were averaged into a mean item index (α = 0.881).

#### Mathematics index (10th and 12th grades)

Three questions were repeated on the 10th and 12th grade surveys related to students' perceptions of ability to overcome challenge in mathematics domains. As with the questions on the other indices, responses to these questions were originally coded 1–4; a score of 4 indicates agreement with each statement and higher perception of ability to overcome challenge in mathematics. Scores on each set of questions were averaged into two separate mean item indices, one each for the 10th grade (α = 0.892) and the other for the 12th grade (α = 0.871).

#### Growth mindset

Finally, one question from the base year survey asked students about their level of agreement with a statement related to Dweck's (2000, 2006) concept of growth mindset (whether or not people could learn to be good at mathematics). Because this is a question specific to one theory and not necessarily related to the other mathematics measures identified in the questionnaires, we let it stand alone. As with the other measures of perceived ability under challenge, this variable was coded such that 1 indicated less agreement and 4 indicated more agreement.

#### Control variables

#### Demographic characteristics

Demographic variables included dichotomous variables for gender, race/ethnicity (white, Asian/Pacific Islander, black, Latino, multi-race/ethnic), parents' education (high school degree or less, less than a 4-year degree, 4-year degree, more than a 4-year degree), and family income by quartiles ($0–$35,000 per year, $35,001–$50,000 per year, $50,001–$100,000 per year, $100,001 or more per year). Detailed information on the coding of these variables is available in the Appendix in Supplementary Material.

Table 2 presents descriptive statistics for the sample and indicates that there are more women in the sample (57.4%) compared to men (42.6%)^3^. Additionally, white students constitute the majority of the sample (73.7%), while the rest of the sample consists of 5.0% Asian American/Pacific Islander students, 9.1% each for black and Latino students, and 3.2% multi-race/ethnicity students. About a quarter of the participants in our sample had parents that earned more than a bachelor's degree, and almost 60.0% of the sample had parents that attended some college or earned a bachelor's degree. 16.1% of the sample had parents with a high school diploma or less. Turning to family income, the largest percentage of our sample (40.7%) came from families that earned between $50,001 and $100,000 per year. 18.0% of the sample had families that earned $35,001–$50,000 per year. Finally, about 20.0% of the sample had families that earned either the lowest level of family income (up to $35,000 per year) or the highest level of family income (more than $100,000 per year).

**Table 2:** **Sample descriptive statistics**.

	**Mean**	**SE**	**Min**	**Max**
**DEMOGRAPHIC CHARACTERISTICS**				
**Gender**				
Men	42.6%	1.1	0.0	100.0
Women	57.4%	1.1	0.0	100.0
**Race/Ethnicity**				
White	73.7%	1.1	0.0	100.0
Asian/Pacific Islander	5.0%	0.4	0.0	100.0
Black	9.1%	0.7	0.0	100.0
Latino	9.1%	0.7	0.0	100.0
Multi-race/Ethnicity	3.2%	0.4	0.0	100.0
**Parent Education**				
High school or less	16.1%	0.9	0.0	100.0
Some college	29.9%	1.0	0.0	100.0
Bachelor's degree	29.0%	0.9	0.0	100.0
More than a bachelor's degree	25.0%	1.1	0.0	100.0
**Family Income**				
First quartile ($0–$35,000 per year)	20.2%	0.9	0.0	100.0
Second quartile ($35,001–$50,000 per year)	18.0%	0.9	0.0	100.0
Third quartile ($50,001–$100,000 per year)	40.7%	1.0	0.0	100.0
Fourth quartile (more than $100,000 per year)	21.1%	1.0	0.0	100.0
**STUDENT ABILITY**				
**Ability With Complex Material**				
Mathematics (10th grade)	2.1%	0.2	0.0	100.0
Reading (10th grade)	16.0%	0.6	0.0	100.0
Grade point average (10th grade)	3.0	0.0	0.4	4.0
**INSTITUTIONAL EFFECTS**				
**HS Region**				
Northeast	20.4%	1.3	0.0	100.0
Midwest	28.4%	1.3	0.0	100.0
South	30.9%	1.3	0.0	100.0
West	20.3%	1.2	0.0	100.0
**HS Urbanicity**				
Urban	27.3%	1.3	0.0	100.0
Suburban	54.4%	1.4	0.0	100.0
Rural	18.4%	1.1	0.0	100.0
**Institutional Selectivity**				
2-year or less institution	27.3%	1.2	0.0	100.0
4-year institution, inclusive	13.4%	0.8	0.0	100.0
4-year institution, moderately selective	30.9%	1.0	0.0	100.0
4-year institution, highly selective	28.4%	1.2	0.0	100.0

n = 4450 respondents from the National Center for Education Statistics' Education Longitudinal Study 2002/2006 restricted data. Student-level replicate weights particular to the base year through 2nd follow-up (2002/2006) waves were used to enhance the correspondence between sample and population results. Restricted-use NCES data requires rounding these descriptive results to the nearest tenth. Ability with complex material (mathematics) and ability with complex material (reading) variables are reported in mean percentage form to more meaningfully explain their characteristics descriptively. Our multivariate analyses use the original form of the variables (on a 0.0–1.0 point scale, not percentages).

#### Student ability

Students' ability was measured through scores on the most complex standardized mathematics and reading questions and grade point average, both in the 10th grade. Scores on the most complex standardized mathematics and reading questions were measured using a continuous variable ranging from 0.0 to 1.0, representing the probability that students would respond correctly to three of the four questions in each category. We used this original form of the variable in our multivariate analyses for the sake of comparability to other studies on this data, but report a percentage form in Table 2 to meaningfully interpret the descriptive statistics. Tenth grade GPA was also a continuous variable ranging from 0.0 to 4.0.

Because all of our student ability measures are continuous in nature, these scores are reported in means. Using a 0.0–100.0 point scale to increase the interpretability of our descriptive statistics, we can see that the mean probability that our sample could complete the most difficult standardized mathematics questions was 2.1%. This indicates that much of our sample had almost no probability of answering three of the four most complex standardized mathematics questions^4^. On the other hand, our sample fared better on the mean probability score of completing three of the four most difficult standardized reading questions, at an average of 16.0% on a 0.0–100.0 point scale. Finally, our sample had a mean 10th grade GPA of 3.0/4.0.

#### High school context.

To control for students' high school contexts, we included measures of their region and urbanicity. Region is based on high school location and corresponds to Census categories: Northeast, Midwest, South, and West. Urbanicity corresponds to NCES classifications: urban, suburban, and rural. Participants in our sample attending high schools across the U.S., with 30.9% concentrated in the South, 28.4% in the Midwest, and just over 20.0% each in the West and Northeast. 54.4% of our sample attended high schools in suburban areas, while 27.3% and 18.4% attended schools in urban and rural areas, respectively.

#### Institutional selectivity

We also controlled for the institutional selectivity of students' first attended postsecondary institutions as of 2 years after high school. Selectivity is split into four dichotomous categories: 2-year college or less, 4-year institution (inclusive or not classified), 4-year institution (moderately selective), and 4-year institution (highly selective). 27.3% of the sample started at a 2-year institution, while 72.7% started at a 4-year institution. Of that 72.7% who started at a 4-year institution, 13.4% first attended an inclusive, 30.9% a moderately selective, and 28.4% a highly selective college or university.

### Analytic plan

Our first research question is primarily concerned with understanding if there are gendered differences in perceived ability under challenge. Therefore, we calculated the sample means for men's and women's scores on each of the perceptions of ability to overcome challenge variables and used Adjusted Wald Tests to provide us with information about significant differences between the two groups. To address the second research question related to the highest science course taken in high school, we used ordered logistic regressions. Finally, we used multiple logistic regressions to examine the third and fourth research questions related to STEM retention and specific major choice. More details regarding our analyses are presented with our results.

## Results

### Gender and perceived ability under challenge in mathematics

In light of previously cited research indicating differences in boys' and girls' assessments of their abilities, we used sample mean Wald tests to determine whether there were significant gender differences on our measures of perceived ability under challenge. Table 3 reveals that in fact young men and women rate themselves as similarly confident in their abilities under challenge in general (Wald = 1.5; *p* = 0.222) as well as in the verbal domain (Wald = 0.4; *p* = 0.555). In contrast, mean differences *between* women and men were highly significant for each measure of perceived ability under challenge in mathematics. Young men were between 0.1 and 0.4 points above the mean on each measure, while young women fell either at or just below the mean in their perceived ability under mathematics challenge in 10th and 12th grades. Notably, the gap between women's and men's ratings of their perceived ability under challenge is largest on the 10th grade mathematics index (diff = 0.4; Wald = 102.9; *p* = 0.000) and tapers slightly 2 years later (diff = 0.2; Wald = 58.7; *p* = 0.000). This change results primarily from a loss of confidence for young men, who see a 0.2 mean centered point decrease between 10th and 12th grades. Among young women, perceptions of their ability on difficult mathematics do not appear to fluctuate over time. Young women are less inclined to report a growth mindset than are young men (diff = 0.2; Wald = 30.5; *p* = 0.000). Together, these findings suggest that young men are better positioned psychologically to be resilient in the face of mathematics-related setbacks, as compared to their female peers.

**Table 3:** **Perceived ability under challenge (mean centered) by gender, weighted**.

	**Mean**		**SE**		**Range**	
	**Men**	**Women**	**Men**	**Women**	**Min**	**Max**
**NON-MATHEMATICS MEASURES**						
General index (10th grade)	0.1	0.2	0.0	0.0	−1.8	1.2
Verbal index (10th grade)	0.2	0.2	0.0	0.0	−1.7	1.3
**MATHEMATICS MEASURES**						
Growth mindset (10th grade)	0.1	−0.1^***^	0.0	0.0	−2.0	1.0
Mathematics index (10th grade)	0.4	0.0^***^	0.0	0.0	−1.5	1.5
Mathematics index (12th grade)	0.2	0.0^***^	0.0	0.0	−1.5	1.5

n = 4450 respondents from the National Center for Education Statistics' Education Longitudinal Study 2002/2006 restricted data. Wald tests were used to determine the significance of difference between the means for men and women. Student-level replicate weights particular to the base year through 2nd follow-up (2002/2006) waves were used to enhance the correspondence between sample and population results. Restricted-use NCES data requires rounding these descriptive results to the nearest tenth. * p < *0.05*, ** p < *0.01*,

*** p < *0.001*.

### Impacts on science course taking

Next, we turned to the question of how advanced science course taking in high school might be distinctly influenced by perceptions of ability under challenge in general, verbal, and mathematics domains. Given that the lower science pipeline courses are pre-requisites of higher science pipeline courses, and thus are ordinal in nature, we used ordered logistic regressions. The first model included our outcome variable and student demographic characteristics. The second model added student ability, high school context, and institutional selectivity to the variables in the first model. Lastly, the third and final model included all of our predictor variables, including the perceived ability under challenge variables. For simplicity, we present the full model only in Table 4. Proportional odds ratios (OR) represent the ratio of odds for completing the highest level of science coursework in high school as compared to the odds of the other combined outcomes (less rigorous courses). When interpreting OR, values under 1 represent negative relationships, values over 1 represent positive relationships, and values approaching 1 represent relationships with less meaningful significance.

**Table 4:** **Likelihood of advanced science course completion by the end of 12th grade**.

	**Chemistry II and Physics II**	
	**OR**	**SE**
**DEMOGRAPHIC CHARACTERISTICS**		
**Female gender (Reference = male)**	0.764^**^	**0.062**
**Race/Ethnicity (Reference = white)**		
Asian/Pacific Islander	2.438^***^	0.338
Black	0.696^*^	0.126
Latino	0.979	0.135
Multi-race/Ethnicity	1.231	0.255
**STUDENT ABILITY**		
**Ability with Complex Material**		
Mathematics (10th grade)	14.324^**^	11.692
Reading (10th grade)	2.028^***^	0.342
Grade point average (10th grade)	2.184^***^	0.156
**INSTITUTIONAL EFFECTS**		
**HS Region (Reference = Northeast)**		
Midwest	1.026	0.174
South	0.940	0.147
West	0.694^*^	0.114
**HS Urbanicity (Reference = urban)**		
Suburban	0.843	0.102
Rural	0.595^**^	0.111
**PERCEIVED ABILITY UNDER CHALLENGE**		
**Non-mathematics measures**		
General index (10th grade)	1.057	0.064
Verbal index (10th grade)	0.911	0.047
**Mathematics Measures**		
Growth mindset (10th grade)	1.004	0.061
Mathematics index (10th grade)	1.296^***^	0.063
Cut1	9.646^***^	2.599
Cut2	34.210^***^	9.541
*f*-statistic	7.050^***^	
Observations	4450	

n = 4450 respondents from the National Center for Education Statistics' Education Longitudinal Study 2002 restricted data. Student-level replicate weights particular to the base year through 2nd follow-up (2002/2006) waves were used to enhance the correspondence between sample and population results. Reference category includes all levels of science course taking less than Physics II and either advanced biology, chemistry, or physics, to conform to the proportional odds assumption. Parent education and family income were included in the model, but are withheld from this table for space. Full tables available from the authors by request.

* p < 0.05,

** p < 0.01,

*** p < 0.001.

Taking all other factors into account, women have about 24.0% lower odds of completing both Chemistry II and Physics II (OR = 0.76; *p* = 0.001) as compared to men, all else being equal. Race/Ethnicity matters as well, as Asian/Pacific Islander students are considerably more likely to complete these more advanced science courses than are white students (OR = 2.44; *p* = 0.000). Conversely, black students are less likely to complete these courses than are their white peers, although this effect is less significant (OR = 0.70; *p* = 0.047).

Objective measures of ability were also meaningfully significant. Students' academic ability with complex material—as measured by test scores—is highly related to completing the most rigorous science courses. Interestingly, this pattern holds for both mathematics and verbal domains. Recall that these scores refer to students' performance on the most challenging sections of the NCES-administered ability tests. A one percentage point increase in one's complex mathematics ability score—an area where most sample respondents struggled, as noted in Table 2—corresponds to having 14 times higher odds of completing both physics II and chemistry II (OR = 14.32; *p* = 0.001). Moreover, the same magnitude of increase in complex reading ability (OR = 2.03; *p* = 0.000) notably enhances the likelihood of completing these courses, all else being equal, as does earning higher grades in school (OR = 2.18; *p* = 0.000).

Although not a focal dimension of this study, the effects of students' high school institutional contexts are worth noting. Access to advanced chemistry and physics coursework is not uniformly available, as noted earlier in this paper. As such, it may not be surprising that students attending high school in Western states (OR = 0.69; *p* = 0.028) are less likely to complete these courses than are students in the Northeast. Correspondingly, students enrolled in rural high schools are less likely to complete these courses than are students in urban high schools (OR = 0.60; *p* = 0.006).

Turning to our primary independent variables of interest, we were surprised that only one perceived ability under challenge measure significantly predicted completion of the most advanced science courses in high school. The 10th grade mathematics index predicts about a 30.0% increase in the odds of taking the highest science courses in high school, holding all other variables constant (OR = 1.30; *p* = 0.000). In contrast, the OR on the growth mindset variable did not reach significance, implying that students' mindset does not affect high school science pipeline completion. Neither verbal nor domain-general perceived ability under challenge significantly predicted completion of these courses.

We examined a model (not shown) including product-term interactions between each of the perceived ability under challenge variables and gender. None of the resulting interaction terms were significant, thus we chose not to display results in this paper due to space constraints. However, the lack of significance on these interaction terms is notable, and combined with the significant effect of the gender variable, indicates that women are less likely to take these courses, but are not affected differentially by perceptions of ability.

### PEMC and/or biology retention and perceived ability under challenge

Table 5 reports on the results of a multiple logistic regression analysis estimating the likelihood of retention in students' intended major. The reference group is comprised of those who neither intended nor declared PEMC and/or biology majors, compared to those who stayed, left, or were newcomers to PEMC and/or biology fields. As with the science pipeline analysis, we estimated four separate models to understand the movement between intended and declared major. Our first model included demographic characteristics only; the second included student ability, high school context, and institutional selectivity; and the third included the perceived ability under challenge indices. For simplicity, we only report the final model using relative risk ratios (RRR). RRR are interpreted as the ratio of the probability that one outcome category will occur compared to that of the reference category (Borooah, 2002; Vogt, 2005). The basic interpretation of ORs and RRRs are congruent: values under 1 represent negative relationships, values over 1 represent positive relationships, and values approaching 1 represent relationships with less meaningful significance.

**Table 5:** **Retention in self-reported intended major, 2 years after high school**.

	**Never entered PEMC and/or biological sciences majors (reference)**					
	**PEMC and/or biological sciences stayers**		**PEMC and/or biological sciences leavers**		**PEMC and/or biological sciences newcomers**	
	**RRR**	**SE**	**RRR**	**SE**	**RRR**	**SE**
**DEMOGRAPHIC CHARACTERISTICS**						
**Female gender (Reference = Male)**	0.194^***^	**0.034**	0.354^***^	**0.068**	**0.749**	**0.142**
**Race/Ethnicity (Reference = White)**						
Asian/Pacific Islander	0.999	0.263	1.059	0.286	1.425	0.515
Black	3.204^***^	0.940	1.102	0.383	1.777	0.596
Latino	0.920	0.300	1.067	0.319	1.520	0.655
Multi-race/Ethnicity	0.855	0.351	1.423	0.652	1.550	0.672
**STUDENT ABILITY**						
**Ability with Complex Material**						
Mathematics (10th grade)	2.358	1.616	2.311	1.826	2.170	1.828
Reading (10th grade)	0.961	0.288	0.830	0.265	0.770	0.282
**Grade point average (10th grade)**	1.583^**^	**0.218**	**0.913**	**0.129**	1.427^*^	**0.241**
**Science Pipeline Completion (Reference = Chemistry I or Physics I or Less)**						
Chemistry I and Physics I	1.779^*^	0.395	1.374	0.316	1.215	0.302
Chemistry II and Physics II	2.722^***^	0.540	2.578^***^	0.558	2.512^***^	0.586
**PERCEIVED ABILITY UNDER CHALLENGE**						
**Non-Mathematics Measures**						
General index (10th grade)	0.979	0.133	1.051	0.144	1.480^*^	0.241
Verbal index (10th grade)	0.582^***^	0.053	0.701^**^	0.076	0.786	0.105
**Mathematics Measures**						
Growth mindset (10th grade)	1.354^*^	0.177	1.244	0.165	1.062	0.176
Mathematics index (10th grade)	1.617^***^	0.175	1.276^*^	0.154	1.084	0.151
Mathematics index (12th grade)	1.561^***^	0.178	1.239	0.140	1.326^*^	0.148
Constant	0.020^***^	0.010	0.070^***^	0.043	0.012^***^	0.008
*f*-statistic	7.050^***^		7.050^***^		7.050^***^	
Observations	4450		4450		4450	

n = 4450 respondents from the National Center for Education Statistics' Education Longitudinal Study 2002 restricted data. Student-level replicate weights particular to the base year through 2nd follow-up (2002/2006) waves were used to enhance the correspondence between sample and population results. Family income, parent education, ability with complex material, high school region and urbanicity, and institutional selectivity were included in the model, but are withheld from this table for space.

* p < 0.05,

** p < 0.01,

*** p < 0.001.

Comparing PEMC and/or biology stayers, leavers, and newcomers with having never expressing interest in those fields (abstainers), there are two significant findings for women. All else being equal, women have an 80.6% lower risk than men of staying in PEMC and/or biology fields as intended before starting college (RRR = 0.19; *p* = 0.000) vs. not entering these fields at all. While the effect is smaller, gender also predicts attrition from PEMC and/or biology fields. Women have a 64.6% lower risk than men of leaving these fields vs. not entering those fields at all (RRR = 0.35; *p* = 0.000), holding all other factors constant. There was also a significant relationship for one race/ethnicity category. All else being equal, our results show that black participants' risk of staying in a PEMC and/or biology field was 3.2 times higher as compared to white participants (RRR = 3.20; *p* = 0.000), among those who intended to major in that field before enrolling in college.

Lastly, there were notable significant results with respect to our student ability measures and science pipeline completion. While complex mathematics and verbal scores were highly predictive of completing advanced high school science coursework, they are no longer significant with respect to major retention, a more advanced step along the scientific pipeline. A 0.01 point increase in 10th grade GPA increased the risk of staying and entering a PEMC and/or biology field by 58.3% (RRR = 1.58; *p* = 0.001) and 42.7% (RRR = 1.43; *p* = 0.036), respectively, as compared to never entering these fields. Science course completion generated the second highest effect sizes in this model. Completing chemistry II and physics II in high school increased the likelihood of staying, leaving, *and* entering PEMC and/or biology fields by over 2.5 times each (all *p* < 0.001), as compared to never intending nor entering those fields. While the similarity of this effect on multiple outcomes may seem puzzling, it perhaps indicates the centrality of high school science course completion to students' entry to the natural sciences at some point early in college, even if it does not singularly predict persistence.

Looking specifically at measures related to our third research question, we see that all perceived ability under mathematics challenge measures—growth mindset, 10th grade mathematics index, and 12th grade mathematics index—positively and significantly predict staying in PEMC and/or biology fields as intended before entering postsecondary education, net of all other factors. The 10th grade mathematics index has the largest effect size here, predicting a 61.7% increased risk of staying in PEMC and/or biology fields as intended (RRR = 1.62; *p* = 0.000) vs. never having entered those fields, compared to 56.1% for the 12th grade mathematics index (RRR = 1.56; *p* = 0.000), and 35.4% for growth mindset (RRR = 1.35; *p* = 0.021). Also consistent with the literature, there is a stronger negative effect on staying in PEMC and/or biology for the verbal index (RRR = 0.58; *p* = 0.000) vs. to leaving (RRR = 0.70; *p* = 0.001), compared to abstaining. However, surprisingly, the 10th grade mathematics index also predicts leaving these fields (RRR = 1.28; *p* = 0.045). Moreover, both the general index and the 12th grade mathematics index predict new entry to PEMC and/or biology fields 2 years after high school (RRR_general index_ = 1.48; *p* = 0.017 and RRR_12th grade mathematics index_ = 1.33; *p* = 0.012). This finding suggests that either domain-general or mathematics-domain perceived ability to overcome challenge might actually encourage students to cross into mathematics-intensive fields of study from non-STEM fields.

Finally, as with the analysis on science pipeline, we examined a model (not shown) including interactions between gender and each of the perceived ability under challenge variables. The resulting coefficients were not significant, so due to space constraints we decided not to show this particular model. Possible explanations for the lack of significance on gender and perceived ability under challenge interaction terms for the major retention variable will be unpacked in the Discussion Section of this paper.

### Specific scientific major and perceptions of ability to overcome challenge

Finally, we examined the relationship between perceived ability under challenge and choice of major 2 years after high school. Since we are primarily interested in how ability-related beliefs might encourage or deter students to major in more mathematics-intensive fields, we disaggregated STEM majors and used non-STEM majors as our reference category. As with the analysis on the major retention variable, we report findings using RRR in Table 6.

**Table 6:** **Specific STEM major category declared 2 years after high school, not including interaction effects**.

**Variables**	**Non-STEM (reference)**									
	**Undeclared/Undecided**		**PEMC**		**Biological sciences**		**Health sciences**		**Soc/Behavioral and other sciences**	
	**RRR**	**SE**	**RRR**	**SE**	**RRR**	**SE**	**RRR**	**SE**	**RRR**	**SE**
**DEMOGRAPHIC CHARACTERISTICS**										
**Female gender (reference = male)**	**0.891**	**0.099**	0.264^***^	**0.048**	**0.953**	**0.206**	3.691^***^	**0.696**	**1.009**	**0.139**
**Race/Ethnicity (reference = White)**										
Asian/Pacific Islander	1.531^*^	0.276	1.113	0.321	2.497^*^	0.904	2.498^**^	0.698	1.423	0.377
Black	1.246	0.332	3.187^***^	1.006	3.394^***^	1.196	1.892^*^	0.504	1.842^*^	0.522
Latino	1.255	0.298	1.340	0.437	1.397	0.703	1.116	0.323	2.088^**^	0.557
Multi-race/Ethnicity	1.041	0.323	1.201	0.486	0.967	0.727	1.680	0.659	0.794	0.367
**STUDENT ABILITY**										
**Science Pipeline Completion (reference = Chemistry I or Physics I or less)**										
Chem. I and Physics I	1.206	0.165	1.850^**^	0.396	1.072	0.331	0.960	0.207	1.030	0.169
Chemistry II and Physics II	1.675^***^	0.258	2.503^***^	0.512	3.875^***^	1.025	1.640^*^	0.314	1.385	0.250
**INSTITUTIONAL EFFECTS**										
**College Selectivity (reference = 4-year institution, highly selective)**										
2-year or less institution	1.131	0.210	0.716	0.181	0.595	0.245	2.409^***^	0.593	0.356^***^	0.087
4-year institution, inclusive	0.399^***^	0.085	0.878	0.223	0.440^*^	0.168	1.807^*^	0.486	0.447^**^	0.110
4-year institution, moderately selective	0.587^***^	0.093	0.708	0.132	1.062	0.293	1.231	0.287	0.728	0.123
**PERCIEVED ABILITY UNDER CHALLENGE**										
**Non-Mathematics Measures**										
General index (10th grade)	1.035	0.090	1.023	0.153	1.583^**^	0.269	1.315^*^	0.173	1.046	0.126
Verbal index (10th grade)	0.990	0.078	0.629^***^	0.064	0.838	0.122	0.808^*^	0.082	1.280^*^	0.131
**Mathematics Measures**										
Growth mindset (10th grade)	0.993	0.091	1.340^*^	0.173	0.953	0.159	1.052	0.104	1.002	0.109
Mathematics index (10th grade)	1.054	0.087	1.599^***^	0.187	1.030	0.125	1.014	0.097	1.027	0.089
Mathematics index (12th grade)	1.014	0.069	1.542^***^	0.174	1.259	0.164	1.054	0.096	0.948	0.091
Constant	0.821	0.349	0.047^***^	0.026	0.014^***^	0.009	0.031^***^	0.017	0.233^**^	0.115
*f*-statistic	9.930^***^		9.930^***^		9.930^***^		9.930^***^		9.930^***^	
Observations	4450		4450		4450		4450		4450	

n = 4450 respondents from the National Center for Education Statistics' Education Longitudinal Study 2002/2006 restricted data. Student-level replicate weights particular to the base year through 2nd follow-up (2002/2006) waves were used to enhance the correspondence between sample and population results. Parent education, family income, ability with complex material, 10th grade GPA, high school region and high school urbanicity were included in the model, but are withheld from this table for space.

* p < 0.05,

** p < 0.01,

*** p < 0.001.

First, we turn to the main effect of gender, which is strongest as a predictor of PEMC and health sciences majors, albeit in opposite directions. While only the third model is shown in Table 6, tables reporting the earlier models are available by request. In the first model, including only demographic characteristics, women have a 0.78 times lower risk than men of majoring in PEMC (RRR = 0.22; *p* = 0.000)^5^ and a 3.59 times higher risk than men of majoring in health (*p* = 0.000), as compared to non-STEM fields. When student ability and institutional effects are added in the second model, women's risk of majoring in PEMC declines slightly as the risk ratio becomes more negative (RRR = 0.20; *p* = 0.000), but their risk of majoring in health does not meaningfully change (RRR = 3.59; *p* = 0.000). The third model adds perceptions under challenge to the model and has a resulting decrease in the negative effect of female gender on the risk of majoring in PEMC (RRR = 0.26; *p* = 0.000) and an increase in the positive effect of female gender on the risk of majoring in health sciences (RRR = 3.69; *p* = 0.000). Adding perceived ability under challenge variables to our models therefore enhances women's chances (relative to men) of majoring in both PEMC and health fields.

Race/ethnicity again plays a role, here influencing declared major 2 years after high school. Holding everything else constant, black students had a 3.19 times higher risk than their white peers of majoring in PEMC as compared to non-STEM fields (*p* = 0.000); they had a 3.39 times higher risk than their white peers of majoring in biology fields (*p* = 0.001). Latinos had a 2.09 times higher risk than their white peers of majoring social/behavioral or other science fields (*p* = 0.006), as compared to non-STEM majors. Asian/Pacific Islander students were at a 2.50 times higher risk than their white peers of majoring in biology (*p* = 0.012) and health (*p* = 0.001), respectively, as compared to non-STEM majors.

Results on student ability and course taking were congruent with the previous analysis of major retention. Tenth grade GPA, net of all other effects, significantly and positively predicted the selection of PEMC majors (RRR = 1.45; *p* = 0.009) and biology majors (RRR = 1.41; *p* = 0.044) vs. non-STEM majors. In contrast, GPA negatively predicted the selection of undeclared/undecided majors (RRR = 0.75; *p* = 0.000), showing that high achieving high school students in our sample tended to select a major by 2 years after high school. Next, the single highest predictor of any major type, holding all other factors constant, was completion of chemistry II and physics II for selection of a biology major (RRR = 3.88; *p* = 0.000). Completion of chemistry II and physics II also increased the risk of enrolling in PEMC fields vs. non-STEM fields (RRR = 2.50; *p* = 0.000), compared to students who only completed chemistry I or physics I or less. Completing even the middle category of the science pipeline variable also benefitted students, predicting an 85.0% increase in the risk of selecting a PEMC major vs. a non-STEM major (*p* = 0.005), as compared to students who only completed chemistry I or physics I and below in high school.

With respect to institutional effects, high school region and college selectivity were the only notable factors influencing choice of major. Students attending high schools in the Midwest and the South were more likely than their peers in the Northeast to select health sciences majors, as compared to non-STEM majors (full table available by request). Attending a less selective institution decreases students' risk of declaring social/behavioral and other science majors, as compared to non-STEM majors. By contrast, their risk of majoring in health sciences increases, in comparison to non-STEM majors. Together, these results suggest that institutional contexts can influence choice of major, in particular health science fields.

Using the product-term regression method (Jaccard and Turrisi, 2003), we can interpret the interactions between gender and perceived ability under challenge measures as slope differences between men and women. In contrast, the main effects for perceived ability under challenge represent the effects of these perceptions for the reference category on gender. Because this manuscript is primarily concerned with how these perceptions influence *women's* entry into scientific majors, we report the results for the case when the reference category for the gender variable is female, so that the main effects of perceived ability under challenge represent the effect for women in particular.

We now turn to the version of the full model shown in Table 7, with women as the reference category and interactions between gender and the perceived ability under challenge variables. Because our perceived ability under challenge variables are mean-centered, a value of 0 refers to the mean value for each of these terms, for the reference category (in this case, women). In this multinomial logistic regression model then, men have a 3.60 times higher risk of majoring in PEMC than women with average perceived ability under challenge (*p* = 0.000) and a 0.74 times lower risk of majoring in health than women with average perceived ability under challenge (*p* = 0.000), again as compared to non-STEM fields. In sum then, holding all other predictors constant, gender strongly influences students' choice of PEMC and health sciences majors. Gender does not however notably influence choice of biological nor social/behavioral and other sciences majors, as compared to non-STEM majors.

**Table 7:** **Specific STEM major category declared 2 years after high school, interaction model**.

	**Non-STEM (reference)**									
	**Undeclared/Undecided**		**PEMC**		**Biological sciences**		**Health sciences**		**Soc/Behavioral and other sciences**	
	**RRR**	**SE**	**RRR**	**SE**	**RRR**	**SE**	**RRR**	**SE**	**RRR**	**SE**
**DEMOGRAPHIC CHARACTERISTICS**										
Male gender (Reference = female)	1.085	0.122	3.604^***^	0.806	1.116	0.286	0.257^***^	0.050	1.032	0.162
**PERCEIVED ABILITY UNDER CHALLENGE**										
**Non-Mathematics Measures**										
General index (10th grade)	1.093	0.137	1.118	0.255	1.746^*^	0.417	1.351^*^	0.193	1.233	0.189
Verbal index (10th grade)	0.894	0.086	0.647^*^	0.119	0.766	0.134	0.762^*^	0.089	1.266	0.166
**Mathematics Measures**										
Growth mindset (10th grade)	1.070	0.134	1.140	0.269	0.891	0.247	1.212	0.136	1.072	0.166
Mathematics index (10th grade)	1.056	0.113	1.360	0.250	1.095	0.176	0.991	0.106	0.950	0.106
Mathematics index (12th grade)	0.931	0.090	1.650^**^	0.285	1.193	0.195	1.040	0.110	0.924	0.109
**INTERACTIONS BETWEEN GENDER AND PERCEIVED ABILITY UNDER CHALLENGE**										
**Non-Mathematics Measures**										
Male*General index (10th grade)	0.884	0.182	0.832	0.223	0.780	0.271	1.029	0.349	0.663	0.142
Male*Verbal index (10th grade)	1.275	0.178	1.003	0.228	1.273	0.354	1.303	0.308	1.037	0.212
**Mathematics Measures**										
Male*Growth mindset (10th grade)	0.844	0.162	1.197	0.332	1.148	0.442	0.505^**^	0.113	0.870	0.187
Male*Mathematics index (10th grade)	0.991	0.155	1.284	0.297	0.850	0.238	1.051	0.257	1.222	0.223
Male*Mathematics index (12th grade)	1.207	0.176	0.934	0.200	1.147	0.304	0.977	0.226	1.056	0.176
Constant	0.730	0.330	0.012^***^	0.007	0.013^***^	0.009	0.114^***^	0.063	0.224^**^	0.116
*f*-statistic	7.790^***^		7.790^***^		7.790^***^		7.790^***^		7.790^***^	
Observations	4450		4450		4450		4450		4450	

n = 4450 respondents from the National Center for Education Statistics' Education Longitudinal Study 2002/2006 restricted data. Student-level replicate weights particular to the base year through 2nd follow-up (2002/2006) waves were used to enhance the correspondence between sample and population results. Family income, parent education, ability with complex material, 10th grade GPA, science pipeline completion, high school region and urbanicity, and college selectivity were included in the model, but are withheld from this table for space.

* p < 0.05,

** p < 0.01,

*** p < 0.001.

**Figure 2:**
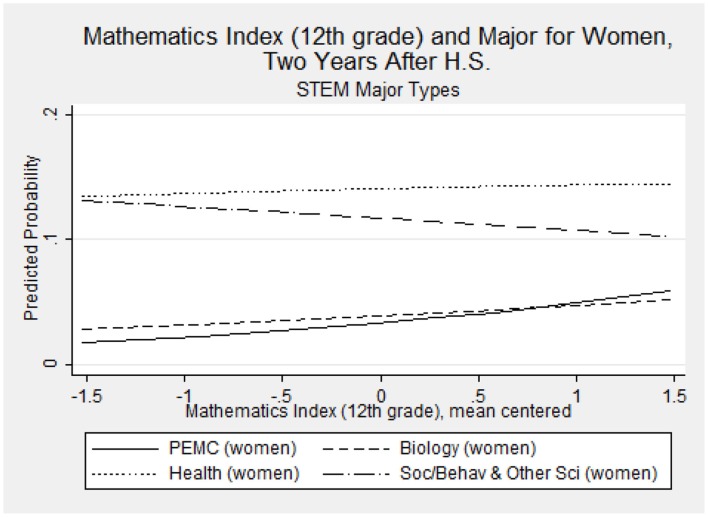
**Mathematics index (12th grade) and probability of majoring in specific STEM majors**.

Recall how in Figure 1, we show our intent to examine how gender moderates the relationship between perceptions of ability under challenge and major choice. Notably, the main effect of the 12th grade mathematics index is the most notable significant perceived ability under mathematics challenge predictor for women, increasing their risk of majoring in PEMC (RRR = 1.65; *p* = 0.004) compared to a non-STEM field^6^. The magnitude and significance of these effects may be somewhat muted, given that there are two indicators in the model for mathematics index (in 10th and 12th grades). This significant result is therefore likely a conservative estimate. To more meaningfully interpret this finding, we used the prgen command from SPost9 (Long and Freese, 2005) to estimate the predicted probabilities for women's selection of each of the major types, given their score on the 12th grade mathematics index. Figure 2 shows the predicted outcomes on a line graph, for each STEM major category. We see that an increase in perceived ability under challenge in mathematics domains meaningfully changes women's probability of declaring PEMC, biology, and social/behavioral and other sciences. Notably, as women's perceived ability increases, their chances of majoring in social/behavioral and other sciences *decreases*. The opposite is true for PEMC and biology. In particular, women's probability of majoring in PEMC increases in association with an increase in their 12th grade perceptions that they could understand and master difficult and complex mathematics material. Specifically, their probability of majoring in PEMC *rises over and above* that of majoring in biology by the point that their perceptions are one unit above the mean for women in our sample.

Rounding out our discussion of how perceived ability under challenge affects women's choice of major, there are two additional findings of note. Domain-general perceptions positively influence the selection of a STEM field in two other instances: biology (RRR = 1.75; *p* = 0.021) and health science fields (RRR = 1.35; *p* = 0.037). Perceived ability in verbal domains also negatively predicts women's entry into PEMC (RRR = 0.65; *p* = 0.019) and health sciences (RRR = 0.76; *p* = 0.020).

**Figure 3:**
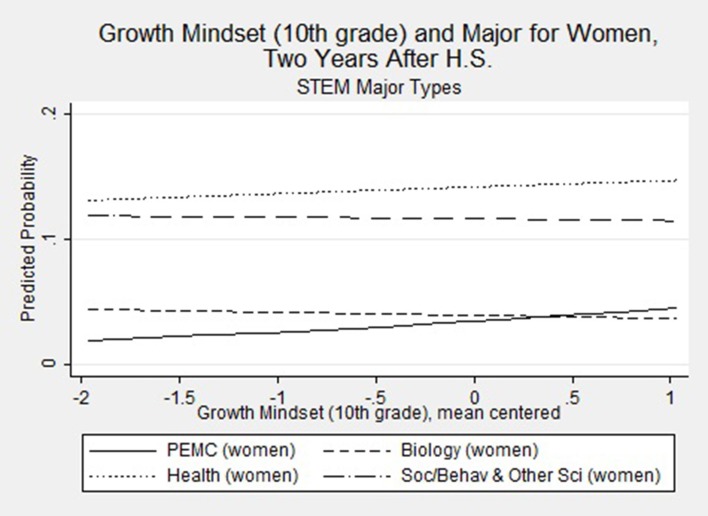
**Growth mindset and probability of majoring in specific STEM majors**.

The interaction terms at the bottom of Table 7 examine the differential impact of gender on perceived ability under challenge. Only one of these interactions is significant in its effect. The male^*^growth mindset interaction term (RRR = 0.51, *p* = 0.003) indicates that gender moderates the effect of growth mindset on students' choice of health science majors as compared to non-STEM majors. This finding indicates that the belief that anyone can improve their mathematics ability through mastery-oriented behavior (growth mindset) differentially effects men and women in a way that promotes women's selection into health science fields. We again use prgen to estimate the predicted probabilities for women's selection of each of the major types, shown in Figure 3, given their score on the growth mindset variable. Consistent with the discussion above, women have both a higher and increasing probability of selecting a health science field as their growth mindset score increases, as compared to the other STEM majors. While the effects are not significant, a sizeable enough increase in growth mindset (a half-point above the mean) appears to positively increase the probability such that—all else held constant—women would have a higher likelihood of majoring in PEMC than they would of majoring in biology. This finding further suggests that there are meaningful, tangible implications for enhancing women's perceptions of their ability under challenge.

## Discussion

### Limitations

Similar to all studies using secondary data sources, our interpretations are limited by the self-reported nature of the data. For instance, our analysis on major retention was limited because students were retrospectively asked the intended major question 2 years after leaving high school. This measure may be biased by their subsequent choice of major. Additionally, this question focused on students' *intent*, not their actual declared major upon entrance into the institutions. While this gives us some insight, declared major symbolizes commitment and would allow us to be reasonably sure that students participated in gateway coursework in the declared major. Further, the coding of the intended major variable did not permit us to disaggregate PEMC fields from biology in the measurement of students' intended majors. As noted by previous researchers, women tend to be overrepresented in biology fields (NSF, 2013), yet we cannot adequately separate out the effects of staying in biology from staying in PEMC fields. Finally, because we do not currently have information on degree completion, our analyses are limited to students' experiences up through 2 years after high school.

## Conclusions

In response to our research questions, we found mixed support for our hypotheses that perceived ability under challenge in mathematics is related to our outcomes of interest: completing advanced science coursework, remaining in intended STEM major fields, and selecting mathematics-intensive science majors (PEMC). Importantly, both gender and perceived ability under challenge in mathematics influence our prediction of all three outcomes. In addition, 10th grade perceptions of ability under challenge in mathematics positively predict completion of the highest levels of high school science coursework. Moreover, all mathematics perceived ability under challenge measures predict both retention in PEMC and/or biology fields, holding all other factors constant. Finally, in some cases, perceptions of ability under challenge affect women's selection of PEMC and other STEM majors.

Turning first to descriptive differences in high school, women and men's perceived ability under challenge differed, with young men in our sample outscoring young women in all perceived ability under mathematics challenge measures. Intriguingly, while the gender gap in perceived mathematics ability seems to taper during high school, this change seems driven by changes among boys rather than girls. Specifically, boys' perceived ability in mathematics decreased between 10th and 12th grade, while girls' perceived ability stayed constant. This finding suggests the need for further empirical and conceptual studies of boys' experiences in mathematics courses in high school, as their relative strengths in this area have been presumed undeserving of examination.

Next, we turn to the predictions of high school course taking. Our results indicate that perceived ability under mathematics challenge in 10th grade matters, and in fact was the only predictive subjective measure (i.e., beyond demographics and ability test scores) of taking advanced science coursework. Female gender negatively predicts advanced science course taking. While recent research suggests that girls are increasingly successful in secondary and postsecondary education, including science course completion (Hill et al., 2010; DiPrete and Buchmann, 2013), our results indicate that gender gaps in course taking remain. However, there were no significant findings for the interaction terms in this analysis, suggesting that something other than perceived ability is at work. Indeed, performance indicators of ability—not perceptions—appeared to particularly influence students' course taking.

Future research may be needed to investigate the mechanisms by which students—girls in particular—are advised into and choose to enroll in a second year of both chemistry and physics, which over 25% of our sample elects to do. These decisions have clear ramifications for entering and choosing PEMC and biology majors, as indicated in our findings reported above. Our negative findings for both western and rural measures of high school location suggest that access to higher-level science coursework is differentially distributed around the U.S. and likely varies by the profiles of students' high schools, not limited to region and urbanicity. For instance, Riegle-Crumb and Moore (2014) show how the density of female STEM professionals in the neighborhoods surrounding schools can mitigate the traditional negative relationship between gender and high school physics course taking. Moreover, recent work by Legewie and DiPrete (2014) on U.S. high school students in the early 1990s indicates that school-level curricular and extra-curricular offerings considerably explain the gender gap in intention to major in STEM at the end of high school. Extensive research and policy initiatives have examined increasing access to advanced mathematics courses. This study suggests that similar attention should be paid to increasing access to advanced science coursework in secondary school, physics in particular.

Despite the number of adequately prepared women entering postsecondary education, we know that fewer of them persist in STEM fields (NSF, 2013). Therefore, we turn next to the matter of how perceived ability under challenge might be related to majoring in PEMC and/or biology as intended at enrollment. As mentioned before, perceived ability under challenge in mathematics (growth mindset, 10th grade mathematics index, and 12th grade mathematics index) is positively related to staying in PEMC and/or biology fields, net of all other effects. This suggests that increasing students' confidence in their ability to deal with difficult mathematics material may lead to retention in those fields. However, there were no significant findings for the interactions between gender and these measures on any level of the retention variable, suggesting that gender does not influence the impact of perceived ability under challenge on retention in students' intended major. These modest results may be the consequence of our limited ability to parse out the PEMC and biology categories^7^, as these STEM fields currently have highly distinct patterns of sex segregation at the undergraduate level, as demonstrated in Table 1.

Mathematics is not the only domain in which perceived ability influences choice of major. As reported in Table 5, we see a significant and negative relationship between perceived ability under challenge in the verbal domain and persistence in PEMC and/or biology. Similarly in Table 7 (and corresponding results in Table 6), perceived ability in verbal domains negatively predicts women's entry into PEMC and health sciences. These findings are consistent with previous literature suggesting that perceived high verbal ability may act as a stronger influence on major choice than actual mathematics ability (Correll, 2001; Wang et al., 2013). Related, we also found that domain-general perceived ability under challenge has a more positive relationship with entering PEMC and/or biology fields than the 12th grade mathematics index, holding all other factors constant. Again, later results on declared majors show that domain-general perceptions positively influence women's selection of biology and health science fields. These results lead us to wonder how domain-general perceived ability may increase interest in certain STEM fields. Future studies, perhaps qualitative in nature, may unpack the mechanisms behind this perhaps puzzling finding.

We were able to disaggregate specific major types in our analysis of the relationships between perceived ability under challenge and declared major 2 years after high school. Results compared across models revealed the specificity of the relationship between gender and each STEM major category. The effects of perceived ability under challenge reported in Table 6 (without interactions) are robust and in the expected direction with respect to the effects on science majors. Also of note are our findings regarding high school region and college selectivity with respect to health fields. With respect to the latter, it is unclear whether it is the institutional context itself or selection into certain institutions that drives the negative relationship between selectivity and health science majors (and correspondingly, the positive relationship between selectivity and social/behavioral and other science majors). As previous research on this topic is inconclusive, this is again an area for potential further investigation.

With respect to the hypothesized moderating effect of gender, the gender-specific results reported in Table 7 did not neatly correspond to our hypotheses. Importantly, we did find an effect for women's selection of a PEMC field when looking at the main effect of the 12th grade mathematics index. Notably, an increase in women's perceived ability with difficult and complex mathematics material increases their probability of majoring in PEMC, such that they become more likely to major in PEMC than in biology. This is notable, as PEMC fields are those that have thus far been the most persistently sex segregated STEM disciplines. As biology and health fields have become more gender egalitarian, and even female-dominant in recent years, this result suggests that interventions aimed at enhancing secondary school girls' perceptions of their mathematics ability can have real effects on their participation in mathematics-intensive fields in postsecondary school, and preventing the loss of scientific talent among young women.

Examining our results on gender moderation further, we found positive gender moderation on the effect of growth mindset for selection into only one STEM field: health sciences. It may be that the intensive and cumulative investment of girls and boys on the scientific pipeline may track those girls with more negative ability-related beliefs out of PEMC fields before they select college majors (Perez-Felkner et al., 2012). Notwithstanding, the effects of perceived ability under challenge for women among mathematics, verbal, and general domains, as well as this finding regarding gender moderation on growth mindset, indicate that there are indeed notable effects to consider and continue to investigate.

Intriguingly, the predicted probabilities shown in Figure 3 indicate that a positive enough mindset among women will increase their probability of majoring in PEMC, even over and above their probability of majoring in biology. Because we did not find significance on the interaction effects between gender and growth mindset on PEMC, we cannot be sure that women who believe that anyone can develop their mathematics ability will enter PEMC majors, a finding seemingly inconsistent with mindset theory (Dweck, 2008; Good et al., 2012; Mangels et al., 2012). It could be that mathematics-intensive fields, such as PEMC, are losing growth mindset women as a result of environmental factors, such as messages that they would fit better or be happier elsewhere (such as the health science fields). These messages may foster stereotype threat.

Stereotype threat occurs when individuals with stereotyped identities fear that they will confirm negative stereotypes (Steele, 1997), and has been widely discussed related to women's choices to leave STEM fields. It is possible that the null findings on most of our interaction terms are masked by the effects of stereotype threat–something we could not directly measure. Although gender did not consistently moderate the relationship between perceived ability under mathematics challenge and our dependent variables, there were strong gender differences in perceptions under challenge across our results, from secondary school through the early postsecondary years. Moreover, while gender did not show a consistent moderating effect, this may be the case for race/ethnicity—a topic beyond the scope of this paper, though no less important to the issue of increasing participation in STEM. Future studies using similar constructs would benefit from additional analyses on the interactive effects of race/ethnicity and perceptions of ability to overcome challenge in pathways to mathematics and science careers.

### Conflict of interest statement

The authors declare that the research was conducted in the absence of any commercial or financial relationships that could be construed as a potential conflict of interest.

## Footnotes

^1^The National Center for Educational Statistics (NCES) released ELS cohort data through 2012 regarding educational attainment. NCES released postsecondary transcript data on this cohort in mid-April, 2015. At the time of this writing, the most up-to-date accurate information regarding majors and degree fields was from the third wave of data, the 2nd follow-up in 2006 (NCES personal communication with authors, 2014).

^2^While we hoped to include Native Americans/Alaskan Natives in our study, they would have comprised only 1.49% of our sample; meaningful results would not have been attainable. We therefore excluded this group from our analysis.

^3^For comparison, according to the U.S. Census Bureau Current Population Surveys, in 2004, the percentage of women enrolled in postsecondary institutions was at 41.2%, exceeding men at 34.7%. As noted in the literature review, women have been exceeding men in postsecondary enrollments in both the U.S. and other industrialized nations, since the 1990s or in some cases earlier. See: http://nces.ed.gov/programs/digest/d13/tables/dt13_302.60.asp

^4^Restricted-use NCES data requires rounding these descriptive results to the nearest tenth.

^5^Note that the effect of gender for men can be found by taking the inverse of these relative ratios. Here, the effect of male gender on the risk of majoring in PEMC is 1/0.22, or 4.55.

^6^The RRR for women, shown in Table 7, is smaller than the result for men (RRR = 1.542; *p* = 0.004), when men are in the reference category (table available by request).

^7^Notably, George-Jackson's (2011) study found that while women persist in PEMC-related fields at lower rates than men, only 24.5% of those who initially chose these majors switched to non-STEM fields; 11.4% switched into biology, health, behavioral, and related science fields. There may be considerable movement among the PEMC and/or biology category that we are not able to observe because of limitations in the coding of the intended major variable.
