# Supplementary material for: Perceived mathematical ability under challenge: a longitudinal perspective on sex segregation among STEM degree fields
Source: Front Psychol. 2015 Jun 9;6:530. doi: 10.3389/fpsyg.2015.00530 (PMC4461727; doi:10.3389/fpsyg.2015.00530)
Supplement: Supplementary file 1 [file DataSheet1.DOCX]

1. **Supplementary Material –** Appendix

**Variable Information**

| **Outcomes** | |
| --- | --- |
|  | *Science pipeline*: NCES-generated variable (F1RSCPIP), collapsed to represent 1 = Chemistry I or Physics I and below; 2 = Chemistry I and Physics I; and 3 = Chemistry II and Physics II. |
|  | *Major retention*: Authors' calculation of whether or not a student stayed in their intended major two years after high school, using NCES-generated variables F2B15 and F2MAJOR2. F2B15 reflected students' response to the question "When you began your post-secondary education, what field of study did you think you were most likely to pursue?" which they answered two years after high school. F2MAJOR2 recorded students' actual majors two years after high school. F2B15 categories and F2MAJOR2 categories differed significantly from one another. In particular, one category for F2B15 combined natural sciences and mathematics, so the authors collapsed all of the categories for both F2B15 and M2MAJOR2 into two categories:   - "PEMC + Bio" (F2B15 categories engineering or engineering technology, computer or information sciences, and natural sciences or mathematics; F2MAJOR2 categories biological and biomedical sciences, computer and information sciences or support technology, engineering technologies and technicians, mathematics and statistics, and physical sciences) and - "All Other Majors."   Major retention was calculated as a comparison between the two variables to reflect   - 1 = PEMC and/or Biology major abstainers (All Other Majors to All Other Majors); - 2 = PEMC and/or Biology stayers (PEMC + Bio to PEMC + Bio); - 3 = PEMC and/or Biology leavers (PEMC + Bio to All Other Majors); and - 4 = PEMC and/or Biology newcomers (All Other Majors to PEMC + Bio). |
|  | *Major type*: Revision of NCES' F2MAJOR2 and F2B22 into the following major categories:   - 1 = Undeclared/Not in a Degree Program (F2B22 categories not in a degree program and not yet declared); - 2 = Non-STEM (F2MAJOR2 categories area, ethnic, cultural, and gender studies; visual and performing arts; business, management, marketing, and related fields; communication, journalism, and communication technology; construction trades; education, English language, literature and letters; family, consumer, and human sciences; foreign languages, literature, and linguistics; legal professions and studies; mechanical and repair technologies and technicians; multi and interdisciplinary studies; parks, recreation, leisure and fitness studies; precision production; personal and culinary services; philosophy, religion and theology; pubic administration and social services; security and protective services; transportation and materials moving; other; and liberal arts, sciences, general studies, and humanities.); - 3 = PEMC (F2MAJOR2 categories computer/info sciences/support tech, engineering technologies/technicians, mathematics and statistics, and physical sciences); - 4 = Biological Sciences (F2MAJOR2 category biological and biomedical sciences); - 5 = Health Sciences (F2MAJOR2 category health professions/clinical sciences); - 6 = Social/Behavioral and Other Sciences (F2MAJOR2 categories agricultural/natural resources/related, architecture and related services, science technologies/technicians, psychology, social sciences). Social/Behavioral sciences and Other STEM collapsed together due to small *n*'s. We included information from F2B22 (whether or not students had declared a major) to avoid dropping students who had not yet declared a major two years after college from the analyses. |
| **Perceptions of Ability to Overcome Challenge** | |
|  | *General index*: An index created from five questions on the 2002 survey (BYS89E, BYS89J, BYS89O, BYS89S, BYS89V) which asked students to rate their level agreement with the following statements: “When I sit myself down to learn something really hard, I can learn it,” “When studying, I keep working even if the material is difficult,” “When studying, I try to work as hard as possible,” “When studying, I try to do my best to acquire the knowledge and skills taught,” and “When studying, I put forth my best effort.” Scores on each of these items ranged from 1 to 4, with higher values representing higher agreement with each statement. The index alpha is 0.861. |
|  | *Verbal index*: An index created from three questions on the 2002 survey (BYS89C, BYS89F, BYS89M) which asked students to rate their level of agreement with the following statements: “I’m certain I can understand the most difficult material presented in English texts,” “I’m confident I can understand the most complex material presented by my English teacher,” and “I’m certain I can master the skills being taught in my English class.” Scores on each of these items ranged from 1 to 4, with higher values representing higher agreement with each statement. The index alpha is 0.880. |
|  | *Growth mindset*: Revision of NCES' BYS88A, which indicated students' agreement with the statement “Most people can learn to be good at math.” The variable was originally coded from 1 to 4 with 1 indicating higher agreement with the statement, therefore we reverse coded it to match the other indices. |
|  | *10th grade math index*: An index created from three questions on the 2002 survey (BYS89B, BYS89L, BYS89U) which asked students to rate their level of agreement with the following statements: “I’m certain I can understand the most difficult material presented in math texts,” “I’m confident I can understand the most complex material presented by my math teacher,” and “I’m certain I can master the skills being taught in my math class.” Scores on each of these items ranged from 1 to 4, with higher values representing higher agreement with each statement. The index alpha is 0.906. |
|  | *12th grade math index*: An index created from three questions on the 2004 survey (F1S18B, F1S18C, F1S18E) which asked students to rate their level of agreement with the following statements: “I’m certain I can understand the most difficult material presented in math texts,” “I’m confident I can understand the most complex material presented by my math teacher,” and “I’m certain I can master the skills being taught in my math class.” Scores on each of these items ranged from 1 to 4, with higher values representing higher agreement with each statement. The index alpha is 0.887. |
| **Demographics** | |
|  | *Gender (female)*: Authors' recalculation of NCES variable BYSEX indicating students' selected sex/gender in 2002. 0 = Men and 1 = Women.  *Gender (male)*: Authors' recalculation of NCES variable BYSEX indicating students' selected sex/gender in 2002. 0 = Women and 1 = Men. |
|  | *Race/Ethnicity*: NCES variable BYRACE_R indicating self-selected race/ethnicity categories asked in 2002. Collapsed and dummy-coded to reflect 1 = White, 2 = Asian/Pacific Islander, 3 = Black, 4 = Latino/Hispanic, 5 = Multi-race/ethnic, 6 = Native American. Native American category excluded from the analysis due to small *n*'s. |
|  | *Parent education*: Revision of NCES variable BYPARED, indicating the highest level of education amongst parents. BYPARED categories were 1 = Did not finish high school; 2 = Graduated from high school or GED; 3 = Attended 2-year school, no degree; 4 = Graduated from 2-year school; 5 = Attended college, no 4-year degree; 6 = Graduated from college; 7 = Completed Master's degree or equivalent; to 8 = Completed PhD, MD, or other professional degree. Our revision collapsed and dummy coded these variables into the following categories: 1 = High school degree or less; 2 = Less than a four-year degree; 3 = A four-year degree; 4 = More than a four-year degree. |
|  | *Family income*: Revised from NCES variable BYINCOME, a 13-level categorical variable indicating family's income level from none to $200,001 or more. The distribution was collapsed and dichotomous-coded by quartile. The first quartile included those receiving no income to those earning $35,000. The next quartile included those receiving $35,001- $50,000 per year. Variable inc_q3 included those making between $50,001 and $100,000 per year. The last quartile included those receiving $100,001 or more per year. |
| **Student Ability** | |
|  | *Ability with complex material, math and reading*: NCES variables BYTX5MPP (math) and BYTX3RPP (reading). These variables were originally generated to allow for comparison between ELS and NELS populations, the probability of proficiency at the highest reading level measures students’ ability to make “Complex inferences or evaluative judgments requiring multiple sources of data” and the probability of proficiency at the highest math level measures students ability with “Complex multi-step word problems and/or advanced mathematical material,” (p. 23, Ingels, Pratt, Rogers, Siegel, and Stutts, 2004). Students were deemed proficient if they correctly answered three of four questions for each category, and both probabilities are measured on a 0-1 scale. |
|  | *10th grade GPA*: NCES variable F1RAGP10, on a 0-4 scale. |
| **Institutional Effects** | |
|  | *HS Region*: A series of four dummy-coded variables derived from NCES variable BYCENDIV. The region variables developed were Northeast, Midwest, South, and West. |
|  | *HS Urbanicity:* Three dummy variables created from NCES variable BYURBAN to form dummy-coded variables that represented urban, suburban, and rural high schools. |
|  | *Institutional selectivity*: Revision of NCES variable F3PS1SLC, to represent the selectivity of students' first postsecondary institution. 1 = Two-year college or less; 2 = Four-year institution (inclusive or not classified); 3 = Four-year institution (moderately selective); and 4 = Four-year institution (highly selective). Values range from 1 to 4 with higher values representing higher levels of selectivity. |
